# Enhanced Water Purification Performance of Ionic Liquid Impregnated Metal–Organic Framework: Dye Removal by [BMIM][PF_6_]/MIL-53(Al) Composite

**DOI:** 10.3389/fchem.2020.622567

**Published:** 2021-01-25

**Authors:** Safiyye Kavak, Özce Durak, Harun Kulak, H. Mert Polat, Seda Keskin, Alper Uzun

**Affiliations:** ^1^Koç University TÜPRAS Energy Center (KUTEM), Koç University, Istanbul, Turkey; ^2^Department of Materials Science and Engineering, Koç University, Istanbul, Turkey; ^3^Department of Chemical and Biological Engineering, Koç University, Istanbul, Turkey; ^4^Koç University Surface Science and Technology Center (KUYTAM), Koç University, Istanbul, Turkey

**Keywords:** composite, water purification, dye removal, ionic liquid, MOF

## Abstract

We incorporated a water-stable ionic liquid (IL), 1-butyl-3-methylimidazolium hexafluorophosphate, [BMIM][PF_6_], into a water-stable metal–organic framework (MOF), MIL-53(Al), to generate the [BMIM][PF_6_]/MIL-53(Al) composite. This composite was examined for water purification by studying its capacity for methylene blue (MB) and methyl orange (MO) removal from aqueous solutions having either single dye or a mixture of both. Data illustrated that the removal efficiency and the maximum adsorption capacity of MIL-53(Al) were increased several times upon [BMIM][PF_6_] incorporation. For instance, within 1 min, 10 mg of pristine MIL-53(Al) adsorbed 23.3% MB from 10 mg/L of MB solution, while [BMIM][PF_6_]/MIL-53(Al) composite was adsorbed 82.3% MB in an identical solution. In the case of MO, 10 mg of pristine MIL-53(Al) achieved 27.8 and 53.6% MO removal from 10 mg/L of MO solution, while [BMIM][PF_6_]/MIL-53(Al) composite removed 61.4 and 99.2% within 5 min and 3 h, respectively. Moreover, upon [BMIM][PF_6_] incorporation, the maximum MB and MO adsorption capacities of the pristine MOF were increased from 84.5 to 44 mg/g to 204.9 to 60 mg/g, respectively. The adsorption of dyes in pristine MIL-53(Al) and [BMIM][PF_6_]/MIL-53(Al) followed a pseudo-second-order kinetic model and a Langmuir isotherm model. In a mixture of both dyes, the IL/MOF composite showed a doubled MB selectivity after the IL incorporation. The composite was successfully regenerated at least two times after its use in water purification to remove MB, MO, and their mixtures. Infrared (IR) spectra indicated that the MB/MO adsorption occurs on [BMIM][PF_6_]/MIL-53(Al) by electrostatic interactions, hydrogen bonding, and π-π interactions. These results showed that [BMIM][PF_6_]/MIL-53(Al) composite is a highly promising material for efficient water purification.

## Introduction

Increased industrialization causes various water pollution issues and evidently gives rise to a global water shortage. Industries such as textile, plastic, paper, food processing, and cosmetics use dyes for coloring purposes and generate effluent wastewater containing various environmentally hazardous dyes (Özcan et al., 2004; Khan et al., 2015). A variety of these dyes can be classified as toxic and carcinogenic in nature, making them hazardous for both the environment and human health (Wang et al., 2005; Mittal et al., 2007; Chen et al., 2010). Moreover, many industrial plants discharge wastewater directly into rivers or oceans, which adversely affect the marine life. Therefore, it is important to efficiently remove and separate synthetic dyes from effluent streams. Hence, efficient water purification strategies should be generated to overcome this issue. In this regard, various types of adsorbent materials have been investigated and reported for efficient dye removal from aqueous solutions, such as activated carbon, coal, clay, fly ash, and metal–organic frameworks (MOFs) (Gupta and Suhas, 2009; Li et al., 2013; Lin et al., 2014; Dias and Petit, 2015; Anastopoulos et al., 2018; Kausar et al., 2018; Arora et al., 2019; Dhaka et al., 2019).

Among these materials, MOFs have excellent structural properties, such as high surface area, high porosity, tunable structure/pore size, unsaturated/saturated metal sites, and thermal/chemical stability (Furukawa et al., 2013). Because of these properties, they have been widely used for many different areas, mainly in gas adsorption and separation, catalysis, drug delivery, photosynthesis, and dye removal from aqueous solutions for water purification purposes (Kavak et al., 2020). For instance, MOF-235 was used for the removal of methylene blue (MB) and methyl orange (MO) from water; and its adsorption capacity for MB and MO was reported as 187 and 477 mg/g, respectively (Haque et al., 2011). MIL-53(Cr) and MIL-101(Cr) along with its ethylenediamine-grafted form, ED-MIL-101(Cr), and its protonated ethylenediamine-grafted form, PED-MIL-101(Cr), were used to remove MO from aqueous solutions as well (Haque et al., 2010). The maximum adsorption capacities of PED-MIL-101(Cr), ED-MIL-101(Cr), MIL-101(Cr), and MIL-53(Cr) were reported as 194, 160, 114, and 57.9 mg/g, respectively. In a recent study, Fe-BDC MOF has been used for the removal of MB from wastewater, and its maximum adsorption capacity was found to be 8.65 mg/g (Arora et al., 2019). They achieved a maximum MB removal of 94.7% from aqueous solutions, having a concentration of 5 mg/L, after 24 h by using 25 mg of Fe-BDC. Molavi et al. (2018) synthesized UiO-66 and used it for the removal of MB and MO from aqueous solutions. The maximum dye adsorption capacities were determined as 69.8 and 83.7 mg/g for MB and MO, respectively. Moreover, Cu-BTC (Lin et al., 2014) and Mn-BTB (He et al., 2018) were used for the removal of MB from aqueous solutions, and their maximum adsorption capacities were reported as 200 and 62.5 mg/g, respectively. The MB adsorption for both MOFs followed a pseudo-second-order kinetic model, and their adsorption isotherms followed the Langmuir isotherm model. Similarly, selective MB adsorption was studied by using an anionic Zn-based MOF (Guo et al., 2017) and a controlled particle-sized Fe-based MOF (Tan et al., 2015), and their maximum adsorption capacities were reported as 348 and 1,105 mg/g, respectively. In the case of an Fe-based MOF (Tan et al., 2015), they explained the adsorption mechanism between MB and MOF by both electrostatic and acid–base interactions.

Beside their adsorption abilities, MOFs have tunable structures, which enable converting them into promising materials toward different applications. They can be modified by the addition of various promoters. In this respect, ionic liquid (IL) incorporation into MOFs, offering novel IL/MOF composites, has become a promising approach for reaching an exceptional performance for various applications, such as gas adsorption and separation (Kinik et al., 2016, 2017; Koyuturk et al., 2017) and water purification (Kavak et al., 2020). For instance, we recently demonstrated that upon incorporating a water-stable IL, [BMIM][PF_6_], into two different water-stable MOFs, UiO-66 and its amine functionalized counterpart NH_2_-UiO-66, an exceptionally rapid dye removal performance could be achieved (Kavak et al., 2020). Motivated by the success of this first demonstration of the use of IL/MOF composites in water purification, here, we extended this approach to a different IL-MOF pair. The same IL, [BMIM][PF_6_], was incorporated into another water-stable MOF, MIL-53(Al), to prepare the [BMIM][PF_6_]/MIL-53(Al) composite. Our recent report demonstrated that this composite is an efficient adsorbent for gas separation applications (Kavak et al., 2019). Considering the same composite for water purification allows us to unravel the extent of opportunities in improving the performance of MOFs for various applications upon the incorporation of ILs. In this respect, we conducted dye removal performance tests using [BMIM][PF_6_]/MIL-53(Al) composite considering both an anionic dye, MO, and a cationic dye, MB, to assess its potential in water purification. Results presented here contribute to the knowledge on the applications of IL/MOF composites in the field of water purification, for which much work is required to reach molecular-level insights on the structure–performance relationships needed for the rational design of new composite materials.

## Materials and Methods

### Materials and Sample Preparation

MIL-53(Al) (Basolite A100, Sigma-Aldrich) was dehydrated overnight under vacuum at 200°C before sample preparation. The IL/MOF composite with 30 wt% IL loading was prepared by using wet impregnation method in open atmosphere (Sezginel et al., 2016). As a first step, 0.3 g of [BMIM][PF_6_] (Sigma-Aldrich) was dissolved in 20 ml of acetone (≥99.5 vol%, Sigma-Aldrich) by stirring at ambient conditions for 1 h. Then, 0.7 g of dehydrated MIL-53(Al) powder was added to the solution. The resulting mixture was stirred in open atmosphere at 35°C to the point where acetone was completely evaporated. The resulting powder, [BMIM][PF_6_]/MIL-53(Al), was dried at 105°C overnight and stored in a desiccator to protect the sample from any impurities and humidity.

### Characterization Techniques

#### X-Ray Fluorescence Spectroscopy

Elemental analysis of pristine MIL-53(Al) and IL/MIL-53(Al) composite was conducted by using a Bruker S8 Tiger spectrometer. An X-ray tube with a 4-kW Rh anode was used, and analysis was performed under helium atmosphere. For data interpretation, SpectraPlus Eval2 V2.2.454 software was utilized.

#### Brunauer–Emmett–Teller Surface Area and Barrett–Joyner–Halenda Pore–Volume Analyses

The N_2_ physisorption analyses were conducted by using Micromeritics TriStar II 3020 analyzer. Prior to measurements, the pristine MIL-53(Al) and [BMIM][PF_6_]/MIL-53(Al) composite were activated for 10 h under vacuum at 150 and 100°C, respectively. Then, the samples were cooled down to −196°C using liquid N_2_, and He gas was used for free-space measurements. Thereafter, N_2_ gas adsorption isotherms were obtained at the pressure range of 10^−6^ to 1 bar at −196°C. The surface area of each sample was estimated by fitting the N_2_ adsorption isotherms to the BET equation by using the relative pressure (*P*/*P*_0_) range from 0.06 to 0.3. By analyzing the obtained adsorption data, pore-size distributions (PSDs) of the samples were calculated with the help of the BJH method.

#### Scanning Electron Microscopy

A Zeiss Evo LS 15 scanning electron microscope (SEM) was used to obtain SEM images of the pristine MIL-53(Al) and [BMIM][PF_6_]/MIL-53(Al) composite. To prevent any issues due to charging, samples were first placed on a carbon tape; and after that, their surfaces were coated with carbon. Analyses were conducted under vacuum with an accelerating voltage of 3 kV, at working distances of 4.1–4.5 mm and magnifications of 100, 50, and 12.5 k ×.

#### X-Ray Diffraction Spectroscopy

X-ray diffraction (XRD) patterns were obtained by using a Bruker D8 Advance instrument that has a Lynxeye detector with a slit width of 1-mm. An X-ray generator that contains a Cu-Kα_1_ radiation source (1.54060 Å) was operated at 30-kV voltage and 10-mA current. The range and the resolution of the analysis were determined accordingly as 2θ at values of 5–90° and 0.0204°, respectively.

#### Fourier Transform Infrared Spectroscopy

Infrared (IR) spectroscopy measurements were performed by using a Bruker Vertex 80v IR spectrometer. For scanning of the background and the sample, scan numbers of 128 and 512 scans were averaged, respectively. Measurements were performed at a spectral resolution of 2 cm^−1^ under vacuum at the wavelength range of 4,000–400 cm^−1^. Pristine MIL-53(Al) and [BMIM][PF_6_]/MIL-53(Al) composite were directly placed between two KBr windows, while the bulk IL was mixed with KBr powder (>99%, purchased from Merck) beforehand. Deconvolution of the obtained IR bands was conducted by using Fityk (Wojdyr, 2010) software with the utilization of Voigt function.

#### X-Ray Photoelectron Spectroscopy

X-ray photoelectron spectroscopy (XPS) measurements were performed by using a Thermo Scientific K-Alpha spectrometer that was equipped with an aluminum anode (Al Kα = 1468.3 eV). The electron take-off angle was set to 90°, and spectra was recorded using an Avantage 5.9 software. The calibration of binding energies was performed by assigning the C 1s signal at 284.8 eV.

#### Thermogravimetric Analysis

A TA Instruments Q500 Thermogravimetric Analyzer with a platinum pan was used for thermogravimetric analysis (TGA) experiments. For pristine MIL-53(Al), bulk IL, [BMIM][PF_6_], and [BMIM][PF_6_]/MIL-53(Al), ~15 mg of sample was placed on the pan prior to the measurement. Thereafter, up to 100°C, a constant temperature ramp rate of 5°C/min was employed; and the samples were treated isothermally for 8 h. After this isothermal treatment, the temperature was increased to 700°C at a ramp rate of 2°C/min. All measurements were conducted under N_2_ flow rates of 40 and 60 ml/min in terms of balance and purge gases, respectively. The onset (*T*_*onset*_) and derivative onset (*T'*_*onset*_) temperatures were determined from the thermogravimetric (TG) and derivative TG curves, respectively, to analyze the thermal stability. The *T'*_*onset*_ values were considered as the temperatures corresponding to the initiation of the thermal decomposition, as *T*_*onset*_ values generally overestimate the thermal decomposition temperatures (Kinik et al., 2016).

### Dye Adsorption Measurements

Both an anionic dye, MO (85%, Sigma-Aldrich), and a cationic dye, MB (≥97%, Sigma-Aldrich), were studied for water purification experiments of [BMIM][PF_6_]/MIL-53(Al) composite. The molecular structures of these dyes are shown in Supplementary Figure 1. For single-dye experiments, 10 mg/L of dye solutions was used, whereas for mixture experiments, 20 mg/L of a dye mixture prepared by mixing equal volume 20 mg/L dye solutions of MO and MB was used.

For dye (MB, MO, and mixture) adsorption studies, 10 mg of adsorbent was immersed into 50 ml of 10 mg/L dye solution. Suspension was mixed by using a Benchmark Scientific Incu-Shaker orbital shaker in a dark environment. After being mixed up to the desired time, the adsorbent was separated from the solution by using Ortoalresa Digicen 21 centrifugation at 5,000 rpm for 5 min. Residual and initial dye concentrations were determined by a Shimadzu UV-3600 UV-Vis-NIR spectrophotometer, using a 10-mm path length quartz cuvette. Data were collected between 800 and 400 nm of wavelength range with medium scan speed that equals to 1-nm sampling interval and 1-nm slit width. The absorbance values of MB and MO were recorded at 664 and 464 nm, respectively, and converted to a concentration by means of a calibration curve. Accordingly, the amount of adsorbed dye in MIL-53(Al) and [BMIM][PF_6_]/MIL-53(Al) composite at equilibrium, *q*_*e*_ (mg/g), as time goes to infinity was calculated according to Equation (1).

(1) $$\begin{array}{r} q_{e}=\frac{V\left(C_{0}-C_{e}\right)}{m} \end{array}$$

where *V* (L) represents the volume of the dye solution, *m* (mg) represents the weight of adsorbent, and *C*_0_ (mg/L) and *C*_*e*_ (mg/L) represent the initial and equilibrium dye concentrations, respectively. Repeated adsorption measurements indicated that the data are consistent within ±5%.

Moreover, the resulting removal efficiencies of MB and MO from their aqueous solutions at a certain time interval were determined by using the obtained initial and final dye concentration values, as follows:

(2) $$\begin{array}{r} R\left(\%\right)=\frac{100\left(C_{0}-C_{t}\right)}{C_{0}} \end{array}$$

where *C*_*t*_ (mg/L) represent the final dye concentration.

To reveal the mechanism of MB and MO adsorptions on pristine MIL-53(Al) and [BMIM][PF_6_]/MIL-53(Al), the experimental data were fitted to both pseudo-first-order and pseudo-second-order kinetic models.

The pseudo-first-order kinetic model equation is as follows:

(3) $$\begin{array}{r} \ln\left(q_{e}-q_{t}\right)=\ln\;q_{e}-k_{1}t \end{array}$$

The pseudo-second-order kinetic model equation is as follows:

(4) $$\begin{array}{r} \frac{t}{q_{t}}=\frac{1}{k_{2}q_{e}^{2}}+\frac{t}{q_{e}} \end{array}$$

where *q*_*e*_ (mg/g) represents adsorbed dye amount at equilibrium, *q*_*t*_ (mg/g) represents adsorbed dye amount at time *t, k*_1_ (1/min) represents the rate constant of pseudo-first-order adsorption kinetics, and *k*_2_ [g/(mg·min)] represents rate constant of pseudo-second-order adsorption kinetics.

Adsorption capacity is an important parameter in addition to the kinetics of dye adsorption for an adsorbent material. To determine the maximum adsorption capacity of MIL-53(Al) and [BMIM][PF_6_]/MIL-53(Al) composite, adsorption experiments with concentrations higher than 10 mg/L (25, 50, 75, and 100 mg/L) of MB and MO in aqueous solutions were performed for 24 h to ensure equilibration. The equilibrium data obtained after each case were fitted to Langmuir (Equation 5) and Freundlich (Equations 6, 7) isotherm models to describe adsorption mechanisms:

(5) $$\begin{array}{r} \frac{C_{e}}{q_{e}}=\;\frac{1}{K_{L}q_{\max}}+\;\frac{C_{e}}{q_{\max}} \end{array}$$

(6) $$\begin{array}{r} q_{e}=K_{f}C_{e}^{1/n} \end{array}$$

The linearized form of Equation (3) can be written as follows:

(7) $$\begin{array}{r} \ln\;q_{e}=\ln\;K_{f}+\frac{1}{n}\ln\;C_{e} \end{array}$$

where *K*_*L*_ (L/mg) represents the Langmuir constant, *q*_*max*_ (mg/g) represents the maximum adsorption capacity, and *K*_*F*_ (mg/g) and *n* (g/L) represent the Freundlich constants.

Regeneration of the adsorbent is another important aspect for the cyclic use of the material in water purification. For the regeneration studies, 10 mg of adsorbent was washed with 40 ml of methanol right after its use in each cycle of mixing and centrifuging. The adsorbent and methanol were loaded into a centrifuge tube and shook by hand for 1 min. Then, it was centrifuged at 5,000 rpm for 5 min; methanol was removed; and cyclic dye adsorption studies were performed with the remaining adsorbent.

## Results and Discussion

By taking advantage of our previous findings demonstrating that the effect of IL on improving the separation performance of MOFs becomes more significant with an increase in IL loading, we incorporated the IL at the highest possible loading before reaching the incipient wetness point (Koyuturk et al., 2017; Kavak et al., 2020). Complete characterization data of this IL-impregnated composite, [BMIM][PF_6_]/MIL-53(Al), along with that of the pristine MOF were already presented in a previous report, where the samples from same batch were considered for gas separation applications (Kavak et al., 2019). In the following paragraph, we briefly present these characterization results.

X-ray fluorescence (XRF) results verified the corresponding [BMIM][PF_6_] loading in the composite as 25.4 ± 1.5 wt.%. The BET surface area and calculated pore volume, which are determined by BJH pore size analysis, for the pristine MOF were 472.7 m^2^/g and 0.189 cm^3^/g, respectively; and the data indicated that these values decrease to 39.6 m^2^/g and 0.059 cm^3^/g, respectively, upon the incorporation of the IL. These decreases indicated that the MOF's pores were partially occupied by the IL molecules. Moreover, COSMO-RS calculations for N_2_ solubility of impregnated bulk IL, [BMIM][PF_6_], at −196°C is given in Supplementary Figure 2. Such poor solubility values can be interpreted as the cause of low BET surface area and pore size analysis of the composite and further prove the presence of IL. The SEM images confirmed that the needle-like crystal structure of MIL-53(Al) remained intact upon the incorporation of IL. Consistently, the XRD results showed that the crystal structure of the MOF was preserved in the composite. Data illustrated that both narrow pores and large pores still co-exist in the framework for both pristine MOF and IL/MOF composite; however, there were slight intensity changes in the spectrum, which indicated the presence of molecular level interactions between the IL and the flexible structure of MIL-53(Al). TGA results further proved the presence of these interactions, which were analyzed in deep detail by IR spectroscopy. Accordingly, red shifts on the band positions of several IL- and MOF-related features, such as ν(C(2)–H), ν_ss_(C(4)HC(5)H), ν_ss_(Al–O–Al), ν_as_(Al–O–Al), δ(O–H), μ_2_(O–H), and ν_as_(PF_6_), were observed. Based on these results, it was inferred that [BMIM][PF_6_] mostly interacts with the aluminum backbone and bridging (O–H) group of MIL-53(Al) (Kavak et al., 2019).

**Figure 1:**
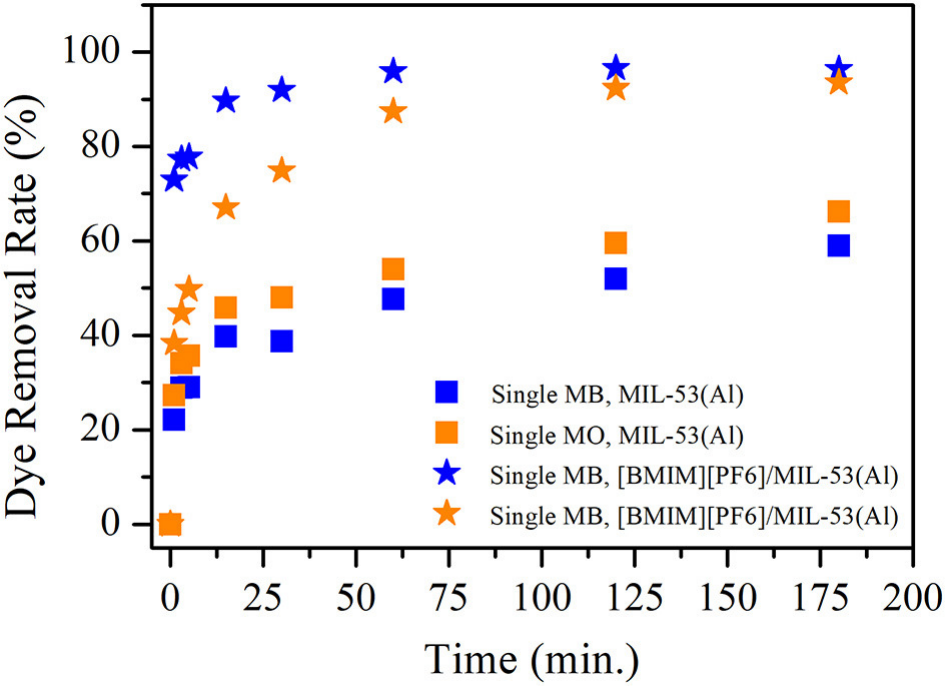
MB and MO removal efficiencies of MIL-53(Al) (squares) and [BMIM][PF_6_]/MIL-53(Al) (stars). Blue (orange) color represents MB (MO) removal for single dye. MB, methylene blue; MO, methyl orange.

Single-component MB and MO removal efficiencies of MIL-53(Al) and [BMIM][PF_6_]/MIL-53(Al) composite are shown in Figure 1. Data illustrated that the dye removal efficiency was enhanced upon the incorporation of IL for both cationic (MB) and anionic dyes (MO). In the case of MB, [BMIM][PF_6_]/MIL-53(Al) adsorbs 82.3% of MB in just 1 min, while MIL-53(Al) achieves only 23.3% MB removal during the same period. Moreover, MB removal efficiency increases to 99.3 and 74.7% for [BMIM][PF_6_]/MIL-53(Al) and MIL-53(Al), respectively, in 3 h. Similarly, in the case of MO, MIL-53(Al) achieves 27.8 and 53.6% MO removal, while [BMIM][PF_6_]/MIL-53(Al) was adsorbing 61.4 and 99.2% of MO in 5 min and 3 h, respectively.

**Figure 2:**
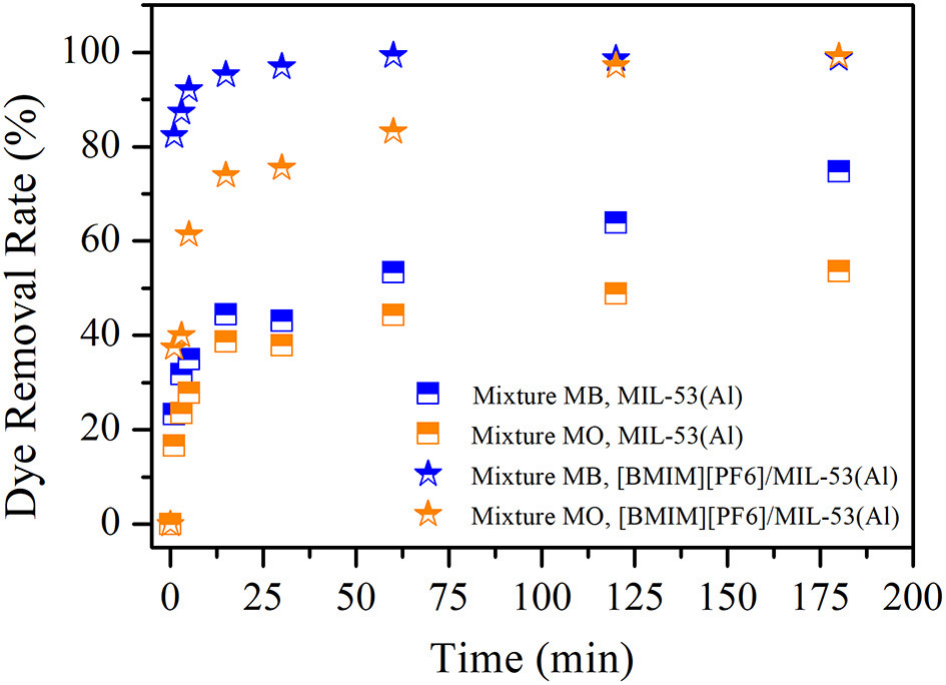
MB and MO removal efficiencies of MIL-53(Al) (squares) and [BMIM][PF_6_]/MIL-53(Al) (stars). Blue (orange) color represents MB (MO) removal for mixture dye. MB, methylene blue; MO, methyl orange.

In Figure 2, removal efficiencies of MB and MO are shown for the case of binary-component MB and MO mixture for both MIL-53(Al) and [BMIM][PF_6_]/MIL-53(Al) adsorbents. The removal efficiencies of MIL-53(Al) and [BMIM][PF_6_]/MIL-53(Al) composite did not show any significant change at single or mixture dye conditions. These results indicate that dyes with different charges do not compete, as the sites responsible for the adsorption of these individual dye molecules are different. Thereby, we infer that [BMIM][PF_6_] incorporation into MIL-53(Al) increases the dye removal efficiency significantly and reduces the time required for reaching the same dye removal efficiency on both MB and MO regardless of single or mixture dye conditions. In addition to these, selective dye adsorption performances of both pristine MIL-53(Al) and [BMIM][PF_6_]/MIL-53(Al) composite were determined by using the measured removal rates for individual dyes in the mixture. According to the results illustrated in Supplementary Figure 3, MB selectivity was doubled in the first 1 to 5 min and then gradually decreased with time upon IL impregnation.

**Figure 3:**
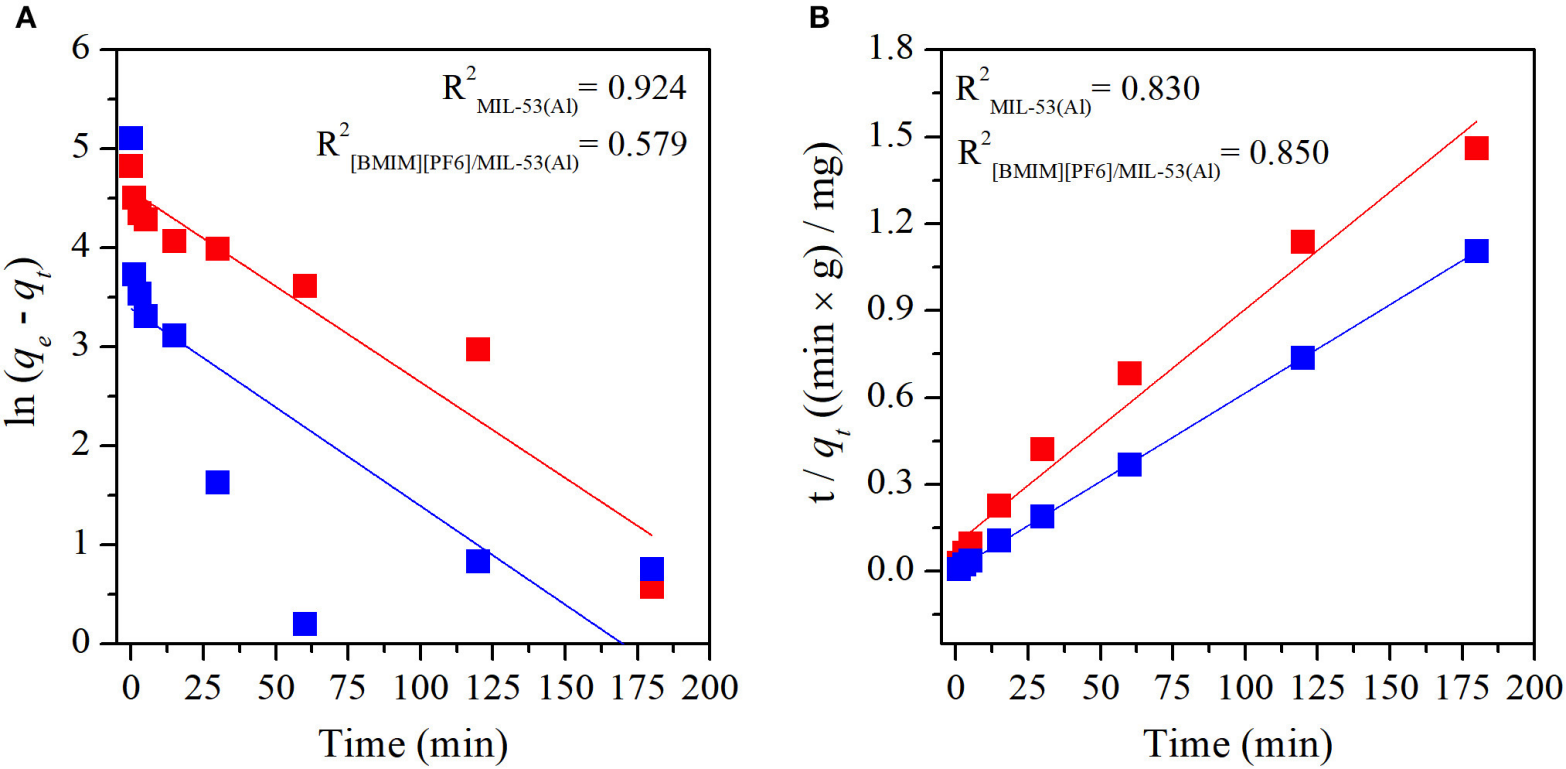
Plots of **(A)** pseudo-first-order and **(B)** pseudo-second-order kinetics of MB adsorption on MIL-53(Al) (red) and [BMIM][PF_6_]/MIL-53(Al) (blue). MB, methylene blue.

**Figure 4:**
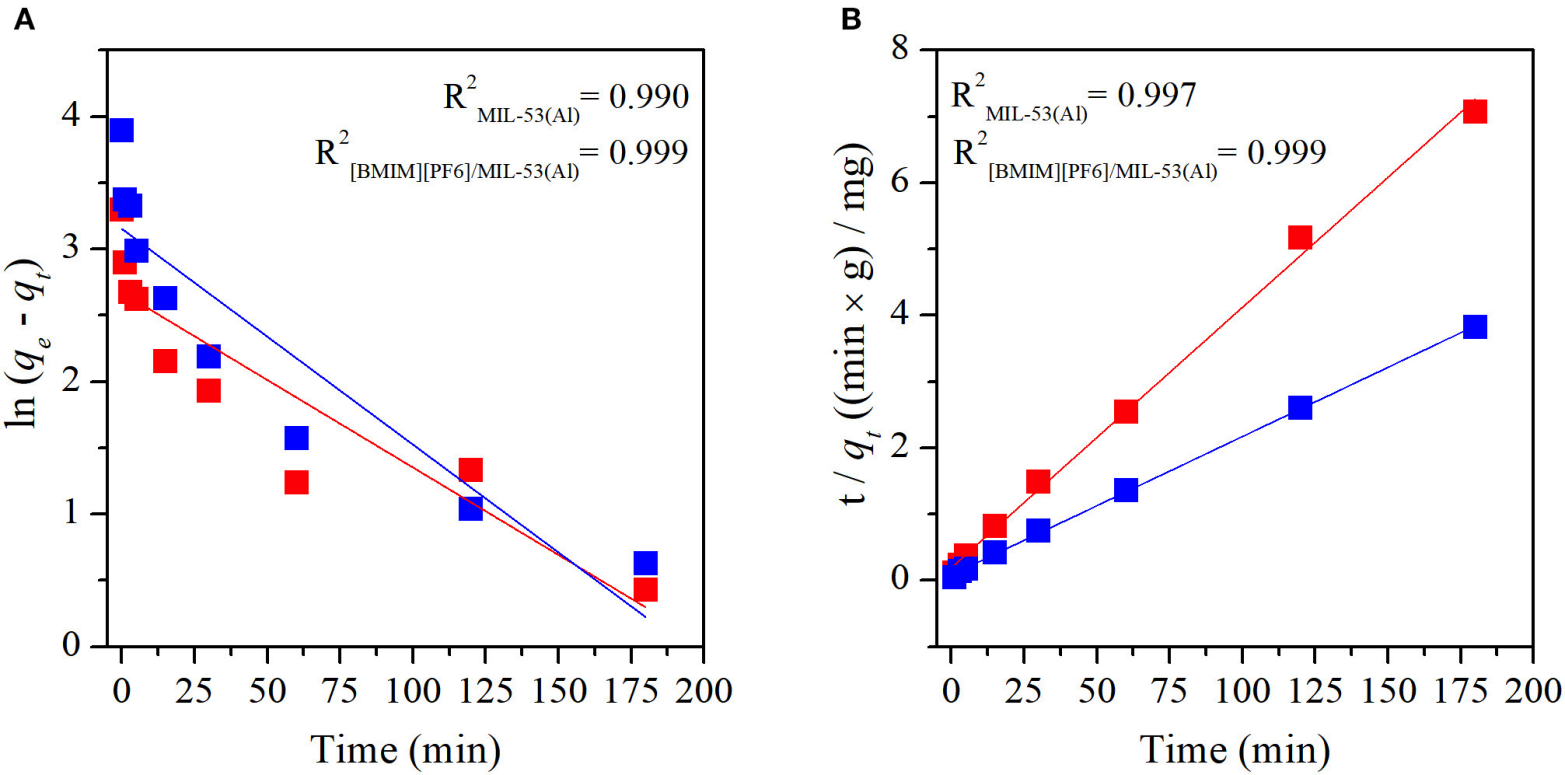
Plots of **(A)** pseudo-first-order and **(B)** pseudo-second-order kinetics of MO adsorption on MIL-53(Al) (red), and [BMIM][PF_6_]/MIL-53(Al) (blue). MO, methyl orange.

Adsorption mechanisms of MB and MO in pristine MIL-53(Al) and [BMIM][PF_6_]/MIL-53(Al) composite were determined by fitting the experimental data to both pseudo-first-order and pseudo-second-order kinetic models. Pseudo-first-order and pseudo-second-order kinetic model fits of MIL-53(Al) and [BMIM][PF_6_]/MIL-53(Al) for MB and MO are given in Figure 3 and Figure 4, respectively. The kinetic parameters of the pseudo-first-order and pseudo-second-order models for both adsorbents are given in Supporting Information, Supplementary Tables 1, 2, respectively. The correlation coefficients (*R*^2^) of the pseudo-second-order kinetic model, 0.980 for MB and 0.999 for MO, were much higher than those of the pseudo-first-order kinetic model, 0.579 and 0.924, respectively, for both materials. The adsorption capacities calculated based on the pseudo-second-order kinetic model (*q*_*e,cal*_) were much closer to the experimentally measured capacities (*q*_*e,exp*_) compared with those calculated based on the pseudo-first-order model, as validated by their higher *R*^2^-values. Therefore, data illustrated that the adsorption of MB and MO in both materials follows the pseudo-second-order model, which is expected because the pseudo-second-order model includes all adsorption processes, such as diffusion through aqueous phase, particle internal diffusion, and the adsorption (Li et al., 2015).

**Figure 5:**
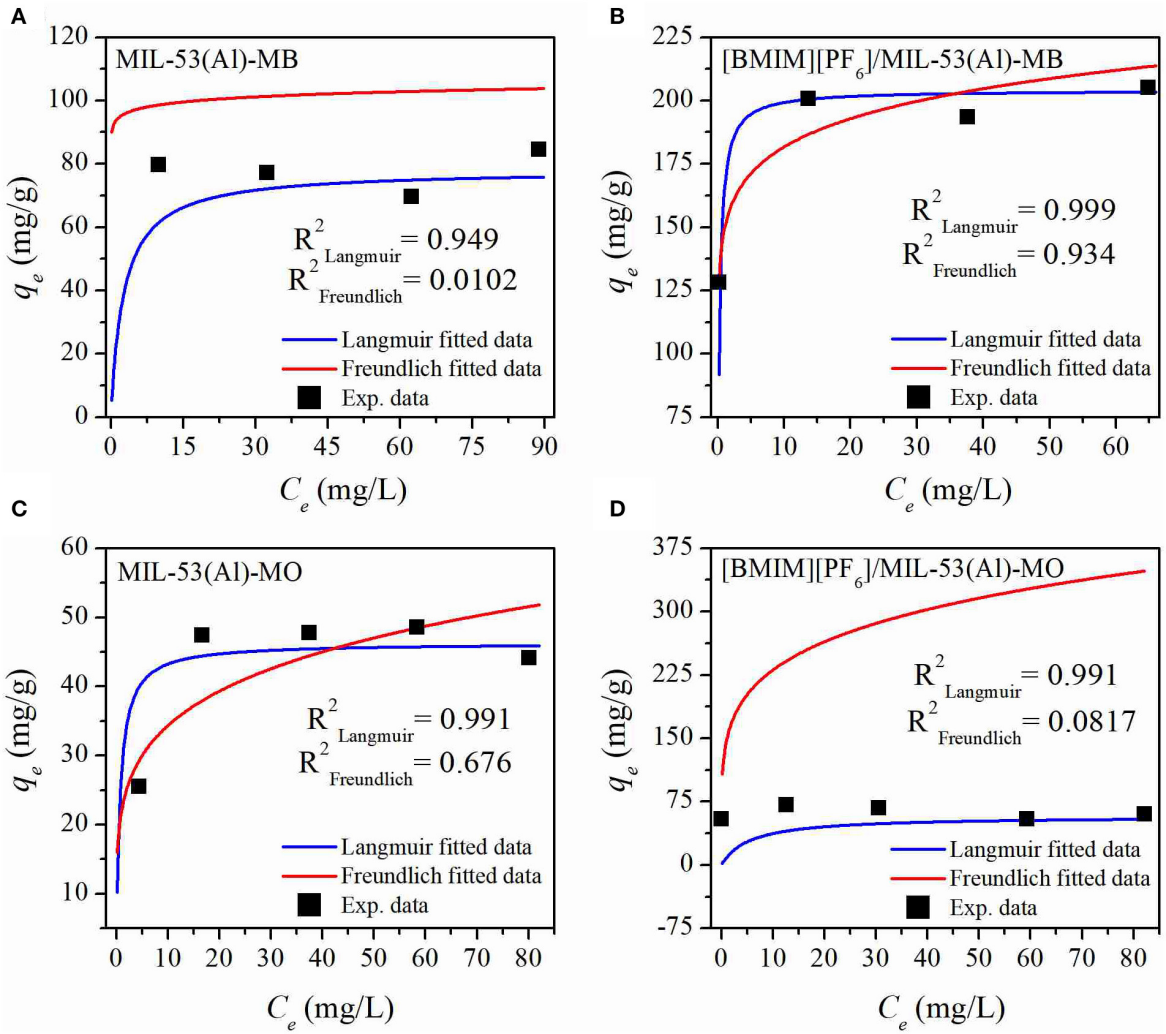
MB **(A,B)** and MO **(C,D)** adsorption isotherms of MIL-53(Al) **(A–C)** and [BMIM][PF_6_]/MIL-53(Al) **(C,D)**. MB, methylene blue; MO, methyl orange.

Maximum dye adsorption capacities of MIL-53(Al) and [BMIM][PF_6_]/MIL-53(Al) were determined according to the results of MB and MO dye adsorption experiments. Consequently, it was obtained that upon [BMIM][PF_6_] incorporation, the maximum MB and MO adsorption capacities of the pristine MOF were increased from 84.5 and 44 mg/g to 204.9 and 60 mg/g, respectively. Moreover, a comparison of the moles of MB adsorbed with the moles of IL present in the IL/MOF composite was made, and it well-justified the results of the adsorption measurements by a ratio of ~1.4 mol of IL for each mole of adsorbed MB. Later on, the equilibrium data were fitted to both Langmuir and Freundlich isotherm models to analyze the adsorption mechanism. The adsorption isotherms are given in Figure 5, and the adsorption isotherm parameters are listed in Supplementary Table 3. As shown in Supplementary Table 3, the correlation coefficients (*R*^2^), above 0.95 for each case, showed that the experimental data have a better fit to the Langmuir model rather than the Freundlich model. The Langmuir model suggests that the adsorption sites are monolayer and assumes that molecules are adsorbed on the adsorbent surface, whereas a multilayer adsorption is suggested, and molecules are non-linearly adsorbed in the Freundlich isotherm model (Lu et al., 2019). Therefore, it can be inferred that the adsorbed dyes, MB and MO, create monolayer adsorption sites on the structure of both pristine and composite materials.

**Figure 6:**
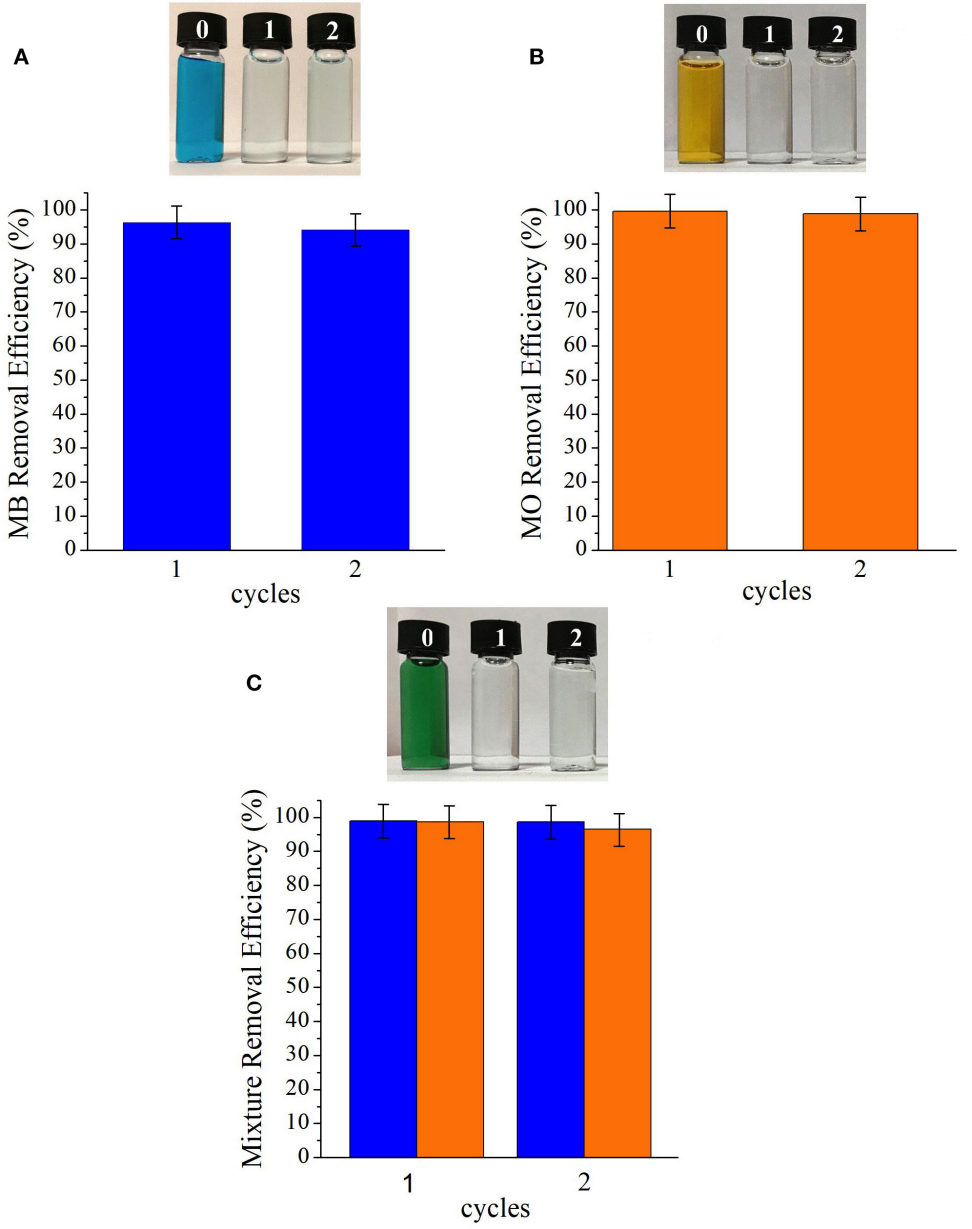
Removal efficiencies of **(A)** MB, **(B)** MO, and **(C)** mixture of MB and MO cycles of [BMIM][PF_6_]/MIL-53(Al). MB, methylene blue; MO, methyl orange.

The adsorbent was regenerated by washing in methanol after usage to evaluate the reusability of the [BMIM][PF_6_]/MIL-53(Al) as an adsorbent material for MB and MO removal from water. Data presented in Figure 6 demonstrate that the composite is reusable without losing its dye removal performance for both dyes. Here, we emphasize that even though the composite can be reused, its recovery without losing any of its mass during its regeneration/washing is crucial.

**Figure 7:**
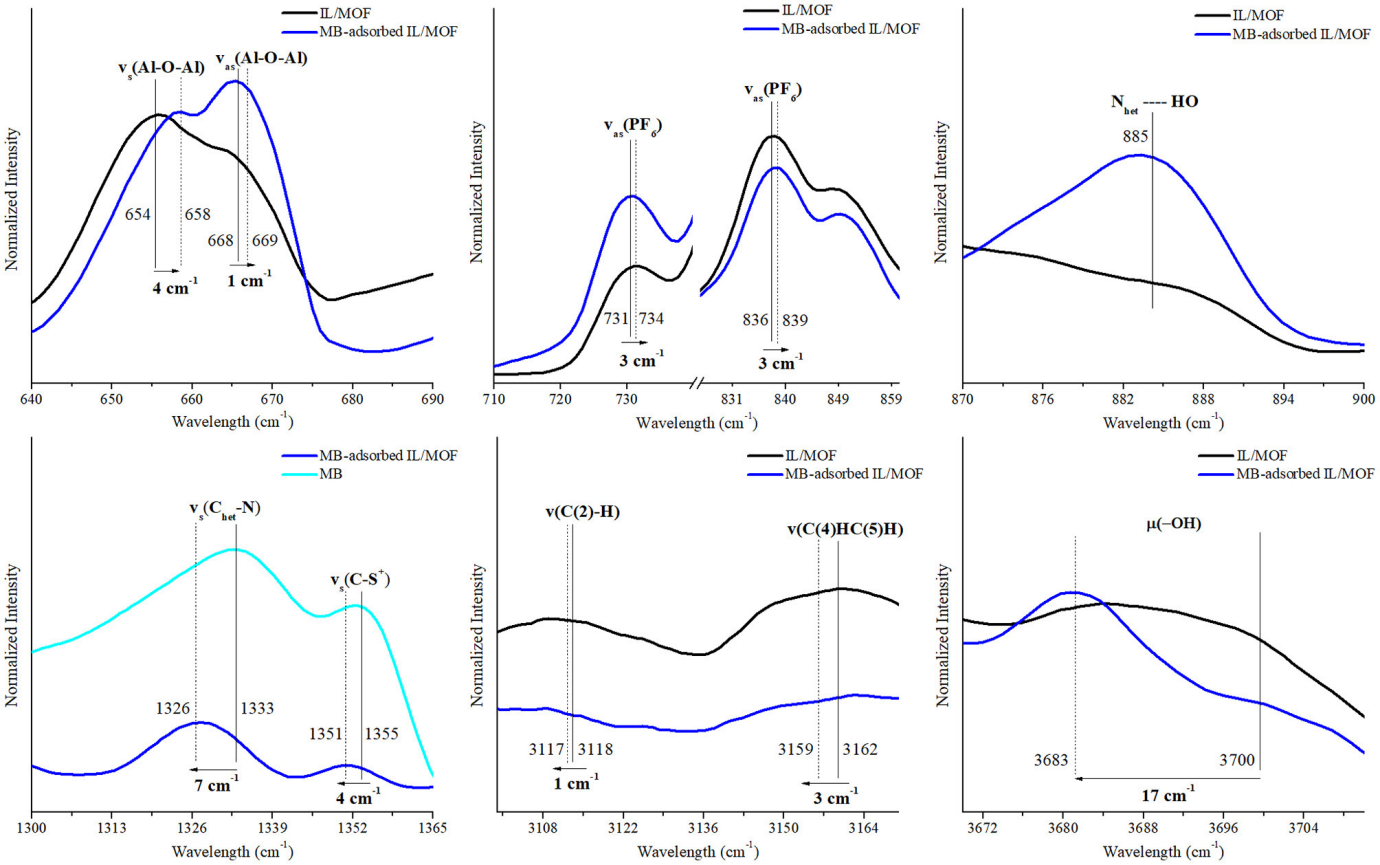
Changes in the characteristic FTIR peaks of [BMIM][PF_6_]/MIL-53(Al) composite and MB after MB adsorption. FTIR, Fourier transform infrared; MB, methylene blue.

**Figure 8:**
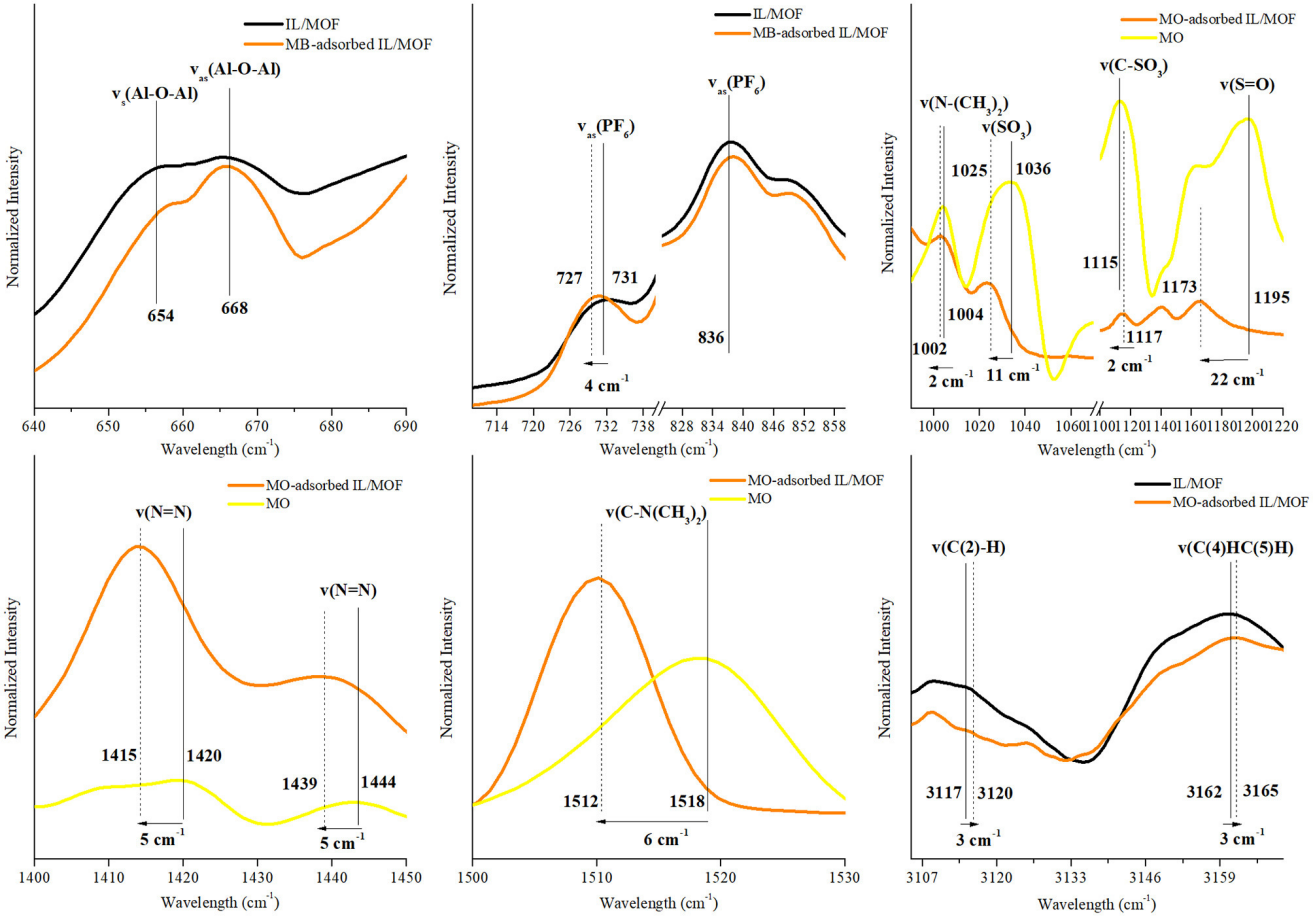
Changes in the characteristic FTIR peaks of [BMIM][PF_6_]/MIL-53(Al) composite and MO after MO adsorption. FTIR, Fourier transform infrared; MO, methyl orange.

**Figure 9:**
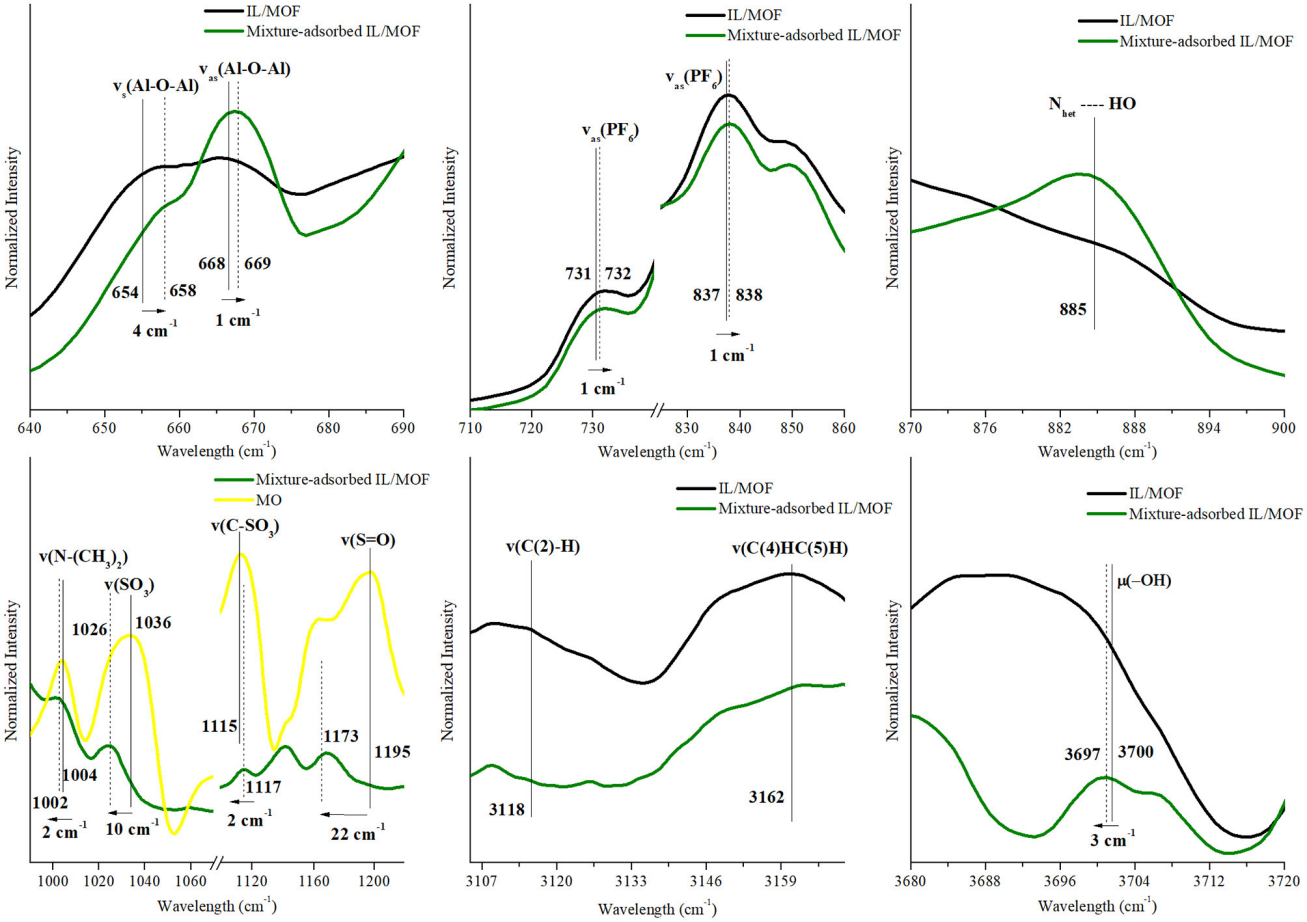
Changes in the characteristic FTIR peaks of [BMIM][PF_6_]/MIL-53(Al) composite, MB, and MO after the adsorption of MB–MO mixture. FTIR, Fourier transform infrared; MB, methylene blue; MO, methyl orange.

To evaluate the possible interaction mechanisms between the [BMIM][PF_6_]/MIL-53(Al) composite, and MB and MO dyes (Hasan and Jhung, 2015; Yoo et al., 2021), IR spectra of the composite before and after dye adsorption were investigated. Supplementary Figure 4 shows that after the adsorption, the characteristic peaks of the dyes (Somani et al., 2003; Raj et al., 2007, Yu and Chuang, 2007; Grumelli et al., 2010; Shen et al., 2015; Ovchinnikov et al., 2016; Grégoire et al., 2019), ν(C–N), ν(C-S^+^), and δ(C–H) for MB and ν(C–H), ν(S-O), and ν(C–N) for MO are present in the IR spectra of dye adsorbed on the [BMIM][PF_6_]/MIL-53(Al) composite for single-component dye case. And as expected, the IR spectrum of [BMIM][PF_6_]/MIL-53(Al) composite measured after the adsorption of the dye mixture has the characteristic peaks of both MB and MO. To identify the interactions between the dye molecules and the composite, we investigated the shifts in the positions of the characteristic features of the individual component of the composite and the dye molecules. The corresponding red or blue shifts amounts determined are presented in Figures 7–9 and in Supplementary Tables 4–7.

According to these results, upon the adsorption of MB onto the composite, symmetric and asymmetric stretching vibrations of the MOF's aluminum backbone, ν_s_(Al–O–Al) and ν_as_(Al–O–Al), presented a blue shift (slightly above the spectroscopic resolution) to higher energy levels by 4 and 1 cm^−1^, respectively, whereas the vibration energy of the bridging O–H groups, μ_2_(O–H), decreased dramatically with a red shift of 17 cm^−1^. These shifts indicate the presence of a direct interaction between the composite and the MB molecules during the adsorption. The nature of this interaction can be identified as hydrogen bonding since a newly formed N_het_…HO peak appeared in the FTIR spectra of MB-adsorbed composite (Ovchinnikov et al., 2016). Moreover, red shifts observed on ν(C_het_-N), ν(C_het_-S^+^), and ν(C_het_-N^+^(CH_3_)_2_) of MB's heterocyclic ring by 7, 4, and 4 cm^−1^, respectively, further support this proposed hydrogen-bonding interaction mechanism between the heterocyclic ring of MB and bridging O–H groups of MIL-53(Al). In addition, asymmetric stretching vibrations of IL's anion, ν_as_(PF_6_), presented a blue shift of 4 and 3 cm^−1^, while ν(C(2)–H), and ν_ss_(C(4)HC(5)H) of IL's cation presented a red shift of 1 and 3 cm^−1^, respectively. Accordingly, it is inferred that the shift in ν_as_(PF_6_) peak can be associated with the interactions between the cationic dye MB and [BMIM][PF_6_]/MIL-53(Al) composite. Cationic dye, MB, can interact with the negatively charged surfaces through electrostatic interactions. Thus, the changes in ν_as_(PF_6_), ν(C(2)–H) and ν_ss_(C(4)HC(5)H) peaks could be attributed to the electrostatic interactions between MB and the anion of IL. Our findings are consistent with the results of a previous report demonstrating the interactions between [BMIM][PF_6_] and cationic dyes in aqueous solutions (Pei et al., 2012).

In contrast with our inference on the MB adsorption mechanism, no changes were observed for the characteristic IR peaks of the MOF upon MO adsorption on the composite. Thus, it can be inferred that there are no interactions between MOF's structure and MO molecules. However, for the characteristic peaks of IL and MO, considerable changes were examined, which can be inferred as an evidence for a possible adsorption mechanism between MO molecules and impregnated IL. In the case of IL related peaks, ν_as_(PF_6_) red shifted by 4 cm^−1^, while ν(C(2)–H) and ν_ss_(C(4)HC(5)H) of IL's cation blue shifted by 3 cm^−1^ and 3 cm^−1^, respectively. Since both [PF_6_]^−^ and MO are negatively charged, the obtained shifts can be attributed to the possible interactions between the cation of IL and MO molecules. Since C(4,5) carbons had π-bonds, such changes in the vibrational energy of ν(C(4,5)H) might be the indicator of π-π interactions between C(4,5) and MO (Matthews et al., 2014). Furthermore, for the case of MO-related IR peaks, red shifts were observed for the stretching vibrations of ν(N–(CH_3_)_2_), ν(SO_3_), ν(S–O), ν(N–N), and ν(C–(CH_3_)_2_) by 2, 11, 2, 5, and 6 cm^−1^, respectively, while only ν(C–SO_3_) blue shifted by 2 cm^−1^. These red shifts indicate the presence of an electron density change in the MO molecule, and they are associated with a possible π-π interaction mechanism between the benzene rings of anionic dye MO and benzene ring of IL's cation. Moreover, blue shift in the stretching vibrations of ν(C–SO_3_) can be inferred as an indicator of a weak electrostatic interaction between the negatively charged surface of MO and positively charged [BMIM]^+^ cation. However, individual contributions of proposed mechanisms cannot be precisely determined due to overlapping aromatic ring peaks of both MO and IL's cation in the FTIR spectra.

For the adsorption of MB–MO mixture, the resulting effects of all proposed mechanisms, for both MB and MO, were observed as excepted. Hydrogen bonding mechanism can be detected by shifts of MOF-related peaks, ν_s_(Al–O–Al), ν_as_(Al–O–Al), and μ_2_(O–H), while a newly formed peak was located around 885 cm^−1^ for N_het_…HO. However, a substantial decrease in the red shift of μ_2_(O–H) was examined. This can be interpreted as the decreasing hydrogen bonding capability of MB molecules in the obtained MB–MO mixture. This result can be associated with the change in the composite's surface electron density with the additional interactions between MO and the IL's cation. Moreover, the effect of π-π interactions and weak electrostatic interactions between MO and IL can also be seen from the shifts of MO-related peaks for mixture-adsorbed IL/MOF composite.

**Figure 10:**
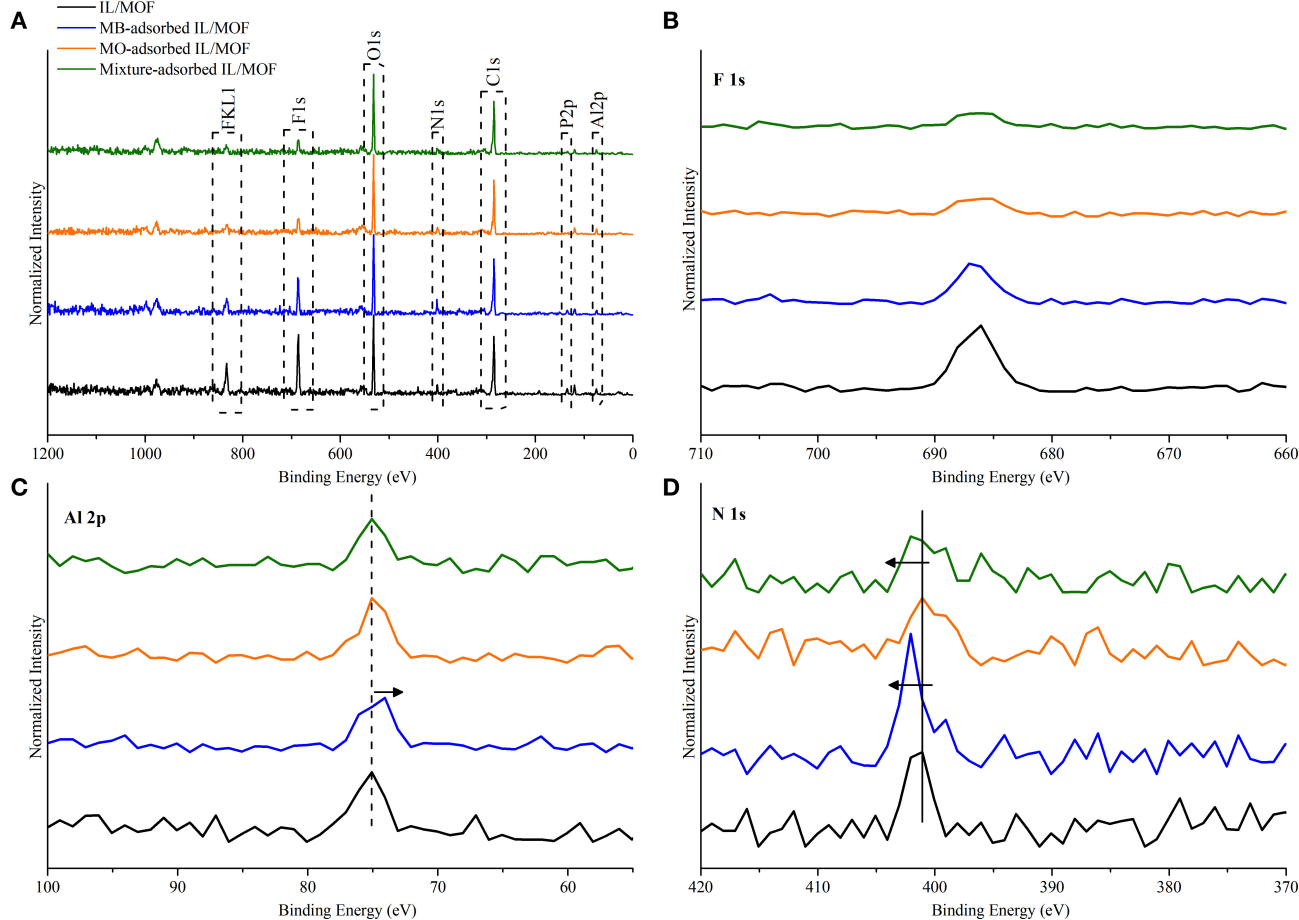
XP spectra of **(A)** IL/MOF composite and IL/MOF composite after the adsorptions of MB, MO, and their mixture; **(B)** F 1s; **(C)** Al 2p; and **(D)** N 1s. XP, X-ray photoelectron; IL, ionic liquid; MOF, metal–organic framework; MB, methylene blue; MO, methyl orange.

To further complement the proposed adsorption mechanisms, XP spectra of the composite material after dye adsorption studies were obtained. As it can be seen in Figure 10, we obtained all the peaks that are related to the MOF and IL's structure as expected. In the case of MOF-related peaks, no shifts were found in Al 2p peak for MO adsorbed composite and MB–MO mixture adsorbed composite; however, a red shift of 0.5 eV was observed in the case of MB adsorbed composite. This shift can be inferred to be an evidence for the direct interactions between MB and MOF's structure as seen in our IR findings. Furthermore, we observed a fall in the features that are mostly related with the anion of IL, PF_6_-, such as F 1s, F KL1, and P 2p. This decrease in the peak features can be attributed to the interaction difference between the MB/MO and the IL, which is also correlated with the interaction strength between MB/MO and IL. As a result, data illustrated a higher decrease in the features of F 1s, F KL1, and P 2p peaks for MO and mixture adsorbed composite proportional to their interactions with the cation of [BMIM][PF_6_]. Moreover, a shift to higher energy levels, 0.4 eV, was examined for N 1s peak of MB adsorbed and MB–MO mixture adsorbed composite, which illustrated the hydrogen bonding between N_het_ of MB molecules and bridging (O–H) of MOF's structure and its resulting effect on N 1s state. The XPS analysis yielded consistent results with our findings of proposed adsorption mechanisms.

In summary, we posit that the adsorption mechanism between MB and the composite consists of hydrogen bonding and electrostatic interactions, while for MO, adsorption mechanism is formed by π-π interactions and weak electrostatic interactions. In addition, for the mixture, the effects of all proposed adsorption mechanisms can be observed. Accordingly, the difference between the maximum adsorption amounts for MO, MB, and their mixture can be attributed to proposed different adsorption mechanisms. The significant improvement of MB adsorption capacity from 84.5 to 204.9 mg/g upon IL incorporation is associated with its hydrogen bonding capability and presence of strong electrostatic interactions between MB's positively charged surface and IL's anion. Furthermore, the maximum adsorption capacity for MO almost did not change after IL incorporation. This slight increase in MO adsorption capacity (44–60 mg/g) can be attributed to the strength of the interaction between IL and MO, mostly π-π interactions, and weak electrostatic interactions in this case. In addition to adsorption capacity enhancement, the MB over MO selectivity, which was doubled upon IL impregnation, can also explained by those strong electrostatic interactions and hydrogen bonding between the IL/MOF composite and MB.

## Conclusion

MB and MO removal performances of [BMIM][PF_6_]/MIL-53(Al), which was identified as the top performer IL/MOF composite for gas separation applications in a previous report (Kavak et al., 2019), were investigated for water purification. Incorporation of [BMIM][PF_6_] into MIL-53(Al) increased its dye removal efficiency and the maximum dye adsorption capacity for both MB and MO. Within 1 min of MB adsorption measurement, IL incorporation increased the MB removal rate of MIL-53(Al) by more than 3.5 times from 23.3 to 82.3% with a starting concentration of 10 mg/L and adsorbent amount of 10 mg. Moreover, MIL-53(Al) achieved 27.8 and 53.6% MO removal, while its IL incorporated counterpart almost doubled the amount adsorbed by adsorbing 61.4% MO in 5 min and 99.2% within 3 h. Upon IL incorporation, maximum MB (MO) adsorption capacity of MIL-53(Al) was increased by more than 2.4 times (1.3 times) from 84.5 (44) mg/g to 204.9 (60) mg/g with an increase in the removal rate for 142.5% (36.4%). In addition, MB/MO selectivity of pristine MIL-53(Al) was doubled after IL incorporation. The adsorption of both MB and MO in MIL-53(Al) and [BMIM][PF_6_]/MIL-53(Al) followed the pseudo-second-order kinetic model; and the adsorption isotherms of both adsorbent for both dyes followed the Langmuir isotherm model. [BMIM][PF_6_]/MIL-53(Al) was successfully regenerated at least two times after the adsorption of MB, MO, and their mixture. Moreover, IR spectra revealed the interaction mechanism between the dyes and [BMIM][PF_6_]/MIL-53(Al) composite as the electrostatic interactions and hydrogen bonding for the case of MB and weak electrostatic interactions and π-π interactions for the case of MO. To conclude, [BMIM][PF_6_]/MIL-53(Al) composite is a highly efficient material for water purification, as it was for gas separation applications. Our results have the potential to lead the way for further studies to select the best IL and MOF couples for the development of new IL/MOF composites that could be path-breaking in the field of water purification.

## Data Availability Statement

The original contributions presented in the study are included in the article/Supplementary Materials, further inquiries can be directed to the corresponding author/s.

## Author Contributions

ÖD, SaK, HK, and HP: conceptualization, investigation, methodology, and writing of the original draft. SeK and AU: supervision, conceptualization, methodology, writing, review, and editing. All authors contributed to the article and approved the submitted version.

## Conflict of Interest

The authors declare that the research was conducted in the absence of any commercial or financial relationships that could be construed as a potential conflict of interest.

## Supplementary Material

Structures of MB and MO, the pseudo-first-order and pseudo-second-order kinetic constants (*k*_1_) together with correlation coefficients (*R*^2^), Langmuir and Freundlich isotherm parameters of MIL-53(Al), and [BMIM][PF_6_]/MIL-53(Al), and FTIR data for MB/MO adsorbed composite.

## References

[B1] Anastopoulos I. Hosseini-Bandegharaei A. Fu J. Mitropoulos A. C. Kyzas Z. G. (2018). Use of nanoparticles for dye adsorption: review. J. Dispers. Sci. Technol. 39, 836–847. 10.1080/01932691.2017.1398661

[B2] Arora C. Soni S. Sahu S. Mittal J. Kumar P. Bajpai K. P. (2019). Iron based metal organic framework for efficient removal of methylene blue dye from industrial waste. J. Mol. Liq. 284, 343–352. 10.1016/j.molliq.2019.04.012

[B3] Chen S. Zhang J. Zhang C. Yue Q. Li Y. Li C. (2010). Equilibrium and kinetic studies of methyl orange and methyl violet adsorption on activated carbon derived from *Phragmites australis*. Desalination 252, 149–156. 10.1016/j.desal.2009.10.010

[B4] Dhaka S. Kumar R. Deep A. Kurade M. B. Ji S.-W. Jeon B.-H. (2019). Metal–organic frameworks (MOFs) for the removal of emerging contaminants from aquatic environments. Coord. Chem. Rev. 380, 330–352. 10.1016/j.ccr.2018.10.003

[B5] Dias E. M. Petit C. (2015). Towards the use of metal–organic frameworks for water reuse: a review of the recent advances in the field of organic pollutants removal and degradation and the next steps in the field. J. Mater. Chem. A 3, 22484–22506. 10.1039/C5TA05440K

[B6] Furukawa H. Cordova K. E. O'Keeffe M. Yaghi M. O. (2013). The chemistry and applications of metal-organic frameworks. Science 341:1230444. 10.1126/science.1230444 23990564

[B7] Grégoire B. Bantignies J.-L. Le-Parc R. Prélot B. Zajac J. Layrac G. (2019). Multiscale mechanistic study of the adsorption of methyl orange on the external surface of layered double hydroxide. J. Phys. Chem. C 123, 22212–22220. 10.1021/acs.jpcc.9b04705

[B8] Grumelli D. Méndez De Leo L. P. Bonazzola C. Zamlynny V. Calvo E. J. Salvarezza R. C. (2010). Methylene blue incorporation into alkanethiol SAMs on Au(111): effect of hydrocarbon chain ordering. Langmuir 26, 8226–8232. 10.1021/la904594p 20356031

[B9] Guo M. Liu S. Guo H. Sun Y. Guo X. Deng R. (2017). The mixed-ligand strategy to assemble a microporous anionic metal–organic framework: Ln3+ post-functionalization, sensors and selective adsorption of dyes. Dalton Trans. 46, 14988–14994. 10.1039/C7DT02506H 29048438

[B10] Gupta V. K. Suhas. (2009). Application of low-cost adsorbents for dye removal – a review. J. Environ. Manage. 90, 2313–2342. 10.1016/j.jenvman.2008.11.017 19264388

[B11] Haque E. Jun J. W. Jhung H. S. (2011). Adsorptive removal of methyl orange and methylene blue from aqueous solution with a metal-organic framework material, iron terephthalate (MOF-235). J. Hazard. Mater. 185, 507–511. 10.1016/j.jhazmat.2010.09.035 20933323

[B12] Haque E. Lee J. E. Jang I. T. Hwang Y. K. Chang J.-S. Jegal J. (2010). Adsorptive removal of methyl orange from aqueous solution with metal-organic frameworks, porous chromium-benzenedicarboxylates. J. Hazard. Mater. 181, 535–542. 10.1016/j.jhazmat.2010.05.047 20627406

[B13] Hasan Z. Jhung S. H. (2015). Removal of hazardous organics from water using metal-organic frameworks (MOFs): plausible mechanisms for selective adsorptions. J. Hazard. Mater. 283, 329–339. 10.1016/j.jhazmat.2014.09.046 25305363

[B14] He J. Li J. Du W. Han Q. Wang Z. Li M. (2018). A mesoporous metal-organic framework: potential advances in selective dye adsorption. J. Alloys Compd. 750, 360–367. 10.1016/j.jallcom.2018.03.393

[B15] Kausar A. Iqbal M. Javed A. Aftab K. Nazli Z.-i.-H. Bhatti H. N. (2018). Dyes adsorption using clay and modified clay: a review. J. Mol. Liq. 256, 395–407. 10.1016/j.molliq.2018.02.034

[B16] Kavak S. Kulak H. Polat H. M. Keskin S. Uzun A. (2020). Fast and selective adsorption of methylene blue from water using [BMIM][PF_6_]-incorporated UiO-66 and NH_2_-UiO-66. Cryst. Growth Des. 20, 3590–3595. 10.1021/acs.cgd.0c00309

[B17] Kavak S. Polat H. M. Kulak H. Keskin S. Uzun A. (2019). MIL-53(Al) as a versatile platform for ionic-liquid/MOF composites to enhance CO_2_ selectivity over CH_4_ and N_2_. Chem. Asian J. 14, 3655–3667. 10.1002/asia.201900634 31339661 PMC6851973

[B18] Khan T. A. Khan E. A. Shahjahan (2015). Removal of basic dyes from aqueous solution by adsorption onto binary iron-manganese oxide coated kaolinite: non-linear isotherm and kinetics modeling. Appl. Clay Sci. 107, 70–77. 10.1016/j.clay.2015.01.005

[B19] Kinik F. P. Altintas C. Balci V. Koyuturk B. Uzun A. Keskin S. (2016). [BMIM][PF_6_] incorporation doubles CO_2_ selectivity of ZIF-8: elucidation of interactions and their consequences on performance. ACS Appl. Mater. Interfaces 8, 30992–31005. 10.1021/acsami.6b11087 27783899

[B20] Kinik F. P. Uzun A. Keskin S. (2017). Ionic liquid/metal–organic framework composites: from synthesis to applications. ChemSusChem 10, 2842–2863. 10.1002/cssc.201700716 28556605

[B21] Koyuturk B. Altintas C. Kinik F. P. Keskin S. Uzun A. (2017). Improving gas separation performance of ZIF-8 by [BMIM][BF_4_] incorporation: interactions and their consequences on performance. J. Phys. Chem. C 121, 10370–10381. 10.1021/acs.jpcc.7b00848

[B22] Li B.-J. Hu J. Huang L.-Y. Lv Y. Zuo J. Zhang W. (2013). Removal of MTBE in biological activated carbon adsorbers. Environ. Prog. Sustain. Energy 32, 239–248. 10.1002/ep.11614

[B23] Li C. Xiong Z. Zhang J. Wu C. (2015). The strengthening role of the amino group in metal–organic framework MIL-53 (Al) for methylene blue and malachite green dye adsorption. J. Chem. Eng. Data 60, 3414–3422. 10.1021/acs.jced.5b00692

[B24] Lin S. Song Z. Che G. Ren A. Li P. Liu C. (2014). Adsorption behavior of metal–organic frameworks for methylene blue from aqueous solution. Microporous Mesoporous Mater. 193, 27–34. 10.1016/j.micromeso.2014.03.004

[B25] Lu J. Zhou Y. Liu Y. (2019). Recent advances for dyes removal using novel adsorbents: a review. Environ. Pollut. 252, 352–365. 10.1016/j.envpol.2019.05.072 31158664

[B26] Matthews R. P. Welton T. Hunt A. P. (2014). Competitive pi interactions and hydrogen bonding within imidazolium ionic liquids. Phys. Chem. Chem. Phys. 16, 3238–3253. 10.1039/c3cp54672a 24407103

[B27] Mittal A. Malviya A. Kaur D. Mittal J. Kurup L. (2007). Studies on the adsorption kinetics and isotherms for the removal and recovery of methyl orange from wastewaters using waste materials. J. Hazard. Mater. 148, 229–240. 10.1016/j.jhazmat.2007.02.028 17379402

[B28] Molavi H. Hakimian A. Shojaei A. Raeiszadeh M. (2018). Selective dye adsorption by highly water stable metal-organic framework: long term stability analysis in aqueous media. Appl. Surf. Sci. 445, 424–436. 10.1016/j.apsusc.2018.03.189

[B29] Ovchinnikov O. V. Evtukhova A. V. Kondratenko T. S. Smirnov M. S. Khokhlov V. Y. Erina V. O. (2016). Manifestation of intermolecular interactions in FTIR spectra of methylene blue molecules. Vib. Spectrosc. 86, 181–189. 10.1016/j.vibspec.2016.06.016

[B30] Özcan A. S. Erdem B. Özcan A. (2004). Adsorption of acid blue 193 from aqueous solutions onto Na–bentonite and DTMA–bentonite. J. Colloid Interface Sci. 280, 44–54. 10.1016/j.jcis.2004.07.035 15476772

[B31] Pei Y. Liu J. Yan Z. Li Z. Fan J. Wang J. (2012). Association of ionic liquids with cationic dyes in aqueous solution: a thermodynamic study. J. Chem. Thermodyn. 47, 223–227. 10.1016/j.jct.2011.10.018

[B32] Raj M. M. Dharmaraja A. Kavitha S. J. Panchanatheswaran K. Lynch E. D. (2007). Mercury(II)–methylene blue interactions: complexation and metallate formation. Inorganica Chim. Acta 360, 1799–1808. 10.1016/j.ica.2006.09.022

[B33] Sezginel K. B. Keskin S. Uzun A. (2016). Tuning the gas separation performance of CuBTC by ionic liquid incorporation. Langmuir 32, 1139–1147. 10.1021/acs.langmuir.5b04123 26741463

[B34] Shen T. Jiang C. Wang C. Sun J. Wang X. Li X. (2015). A TiO_2_ modified abiotic–biotic process for the degradation of the azo dye methyl orange. RSC Adv. 5, 58704–58712. 10.1039/C5RA06686G

[B35] Somani P. R. Marimuthu R. Viswanath A. K. Radhakrishnan S. (2003). Thermal degradation properties of solid polymer electrolyte (polyvinyl alcohol)+phosphoric acid)/methylene blue composites. Polym. Degrad. Stab. 79, 77–83. 10.1016/S0141-3910(02)00240-9

[B36] Tan F. Liu M. Li K. Wang Y. Wang J. Guo X. (2015). Facile synthesis of size-controlled MIL-100(Fe) with excellent adsorption capacity for methylene blue. Chem. Eng. J. 281, 360–367. 10.1016/j.cej.2015.06.044

[B37] Wang S. Boyjoo Y. Choueib A. Zhu Z. (2005). Removal of dyes from aqueous solutions using fly ash and red mud. Water Res. 39, 129–138. 10.1016/j.watres.2004.09.011 15607172

[B38] Wojdyr M. (2010). Fityk: a general-purpose peak fitting program. J. Appl. Crystallogr. 43, 1126–1128. 10.1107/S0021889810030499

[B39] Yoo D. K. Bhadra B. N. Jhung H. S. (2021). Adsorptive removal of hazardous organics from water and fuel with functionalized metal-organic frameworks: contribution of functional groups. J. Hazard. Mater. 403:123655. 10.1016/j.jhazmat.2020.123655 33264864

[B40] Yu Z. Chuang S. S. C. (2007). Probing methylene blue photocatalytic degradation by adsorbed ethanol with *in situ* IR. J. Phys. Chem. C 111, 13813–13820. 10.1021/jp0715474

