# Supplementary material for: Enhanced Water Purification Performance of Ionic Liquid Impregnated Metal–Organic Framework: Dye Removal by [BMIM][PF6]/MIL-53(Al) Composite
Source: Front Chem. 2021 Jan 25;8:622567. doi: 10.3389/fchem.2020.622567 (PMC7868392; doi:10.3389/fchem.2020.622567)
Supplement: Supplementary file 1 [file Data_Sheet_1.PDF]

Supplementary Material

"Enhanced Water Purification Performance of Ionic Liquid Impregnated MOF: Dye Removal by [BMIM][PF6]/MIL-53(Al) Composite"

(A)

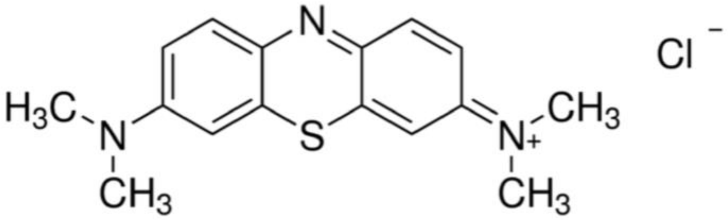

(B)

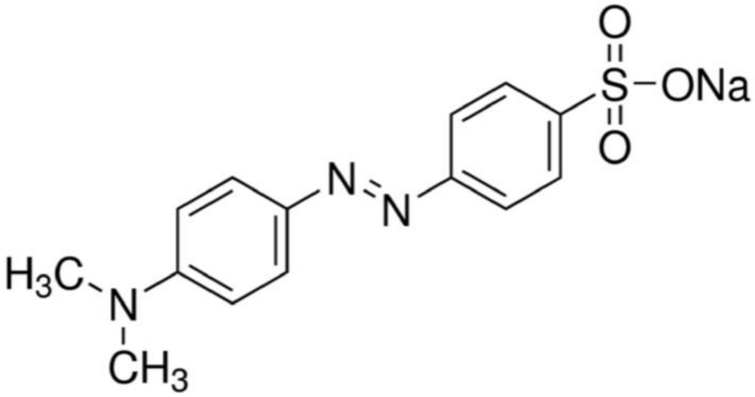

Figure S1. Structure of **(A)** cationic dye methylene blue (MB), **(B)** anionic dye methyl orange (MO)

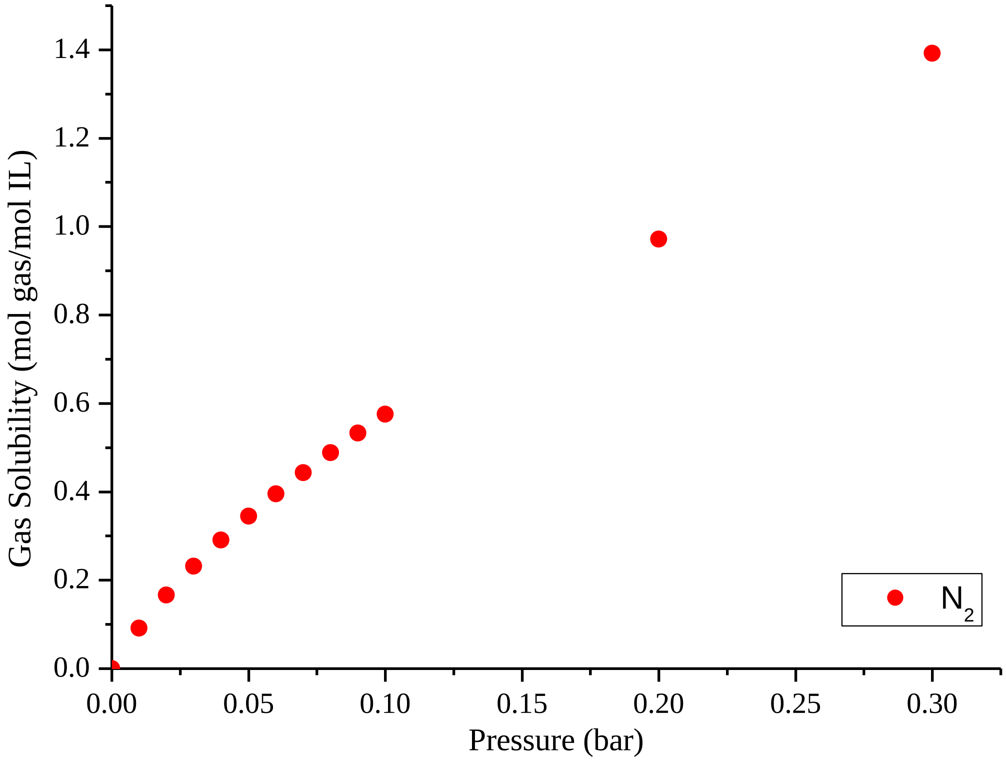

Figure S2. Solubility results of N_2 in bulk [BMIM][PF6] obtained by COSMO-RS calculations.

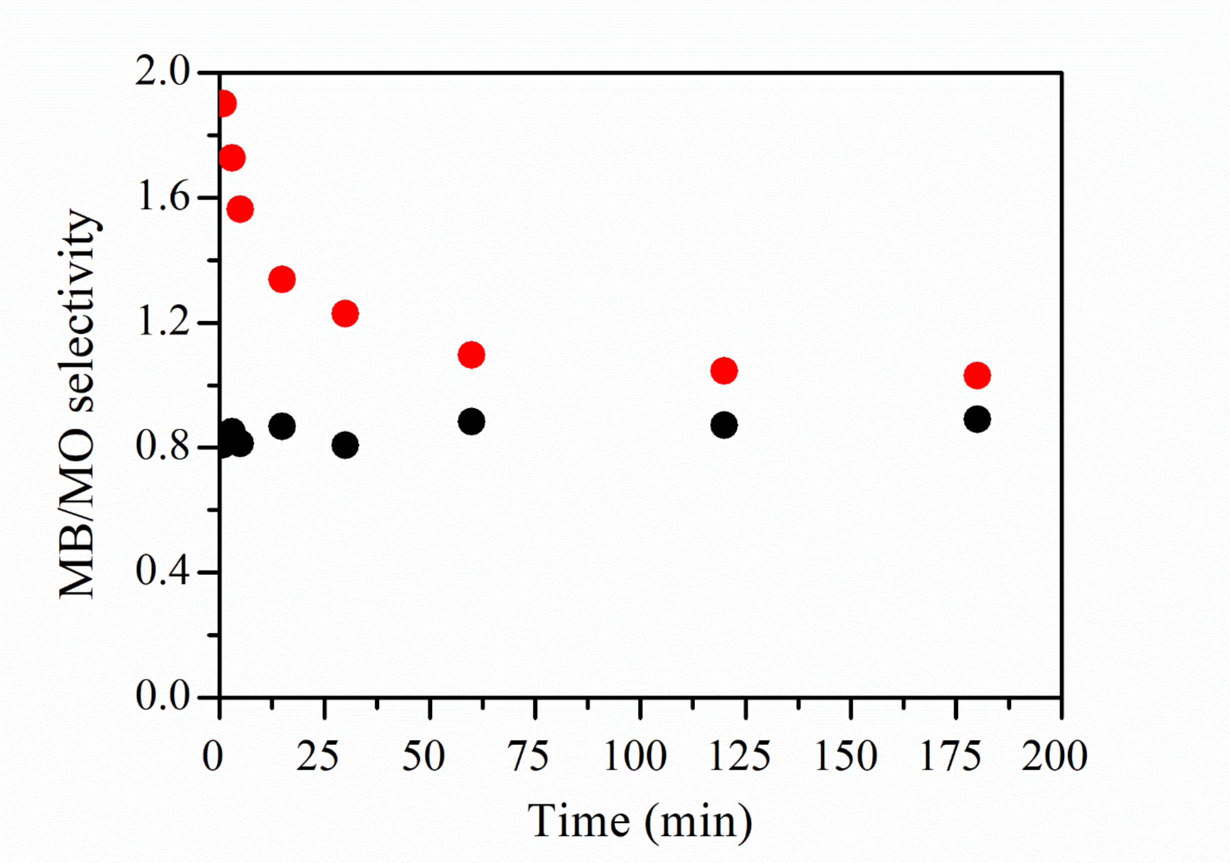

Figure S3. MB/MO removal selectivity of pristine MIL-53(Al) (black) and [BMIM][PF_6]/MIL-53(Al) composite (red).

Table S1. The pseudo-first-order kinetic constants (k_1) together with correlation coefficients (R^2) of MIL-53(Al), and [BMIM][PF_6]/MIL-53(Al).

**Adsorbents**	**Dyes**	$q_{e,exp}$ **(mg/g)**	k_1 **(1/min)**	$q_{e,cal}$ **(mg/g)**	R^2	***RMSE***
**MIL-53(Al)**	MB	123.22	1.94×10^{-2}	97.27	0.924	0.275
	MO	25.46	1.32×10^{-2}	14.45	0.830	0.412
**[BMIM][PF_6]/ MIL-53(Al)**	MB	162.88	1.99×10^{-2}	29.48	0.579	0.649
	MO	47.12	1.63×10^{-2}	20.14	0.850	0.387

Table S2. The pseudo-second-order kinetic constants (k_2) together with correlation coefficients (R^2) of MIL-53(Al), and [BMIM][PF_6]/MIL-53(Al).

**Adsorbents**	**Dyes**	$q_{e,exp}$ **(mg/g)**	k_2 **(g/(mg min))**	$q_{e,cal}$ **(mg/g)**	R^2	***RMSE***
**MIL-53(Al)**	MB	123.22	6.90×10^{-4}	123.46	0.980	0.141
	MO	25.46	9.62×10^{-3}	25.32	0.997	0.055
**[BMIM][PF_6]/ MIL-53(Al)**	MB	162.88	7.02×10^{-3}	163.93	0.999	0.032
	MO	47.12	6.36×10^{-3}	47.62	0.999	0.032

Table S3. Langmuir and Freundlich isotherm parameters of MIL-53(Al), and [BMIM][PF_6]/MIL-53(Al).

			Langmuir Model				Freundlich Model			
**Adsorbents**	**Dye**	$q_{e,exp}$ (mg/g)	K_L (L/mg)	q_{max} (mg/g)	R^2	*RMSE*	K_F (mg/g)	*n* (g/L)	R^2	*RMSE*
**MIL-53(Al)**	MB	84.546	0.371	78.125	0.949	0.226	93.504	43.290	0.0102	0.995
	MO	44.089	1.421	46.296	0.991	0.095	21.909	5.120	0.676	0.569
**[BMIM][PF_6]/ MIL-53(Al)**	MB	204.905	4.083	204.082	0.999	0.032	149.038	11.628	0.934	0.257
	MO	60.283	0.182	57.803	0.991	0.095	147.496	5.133	0.0817	0.958

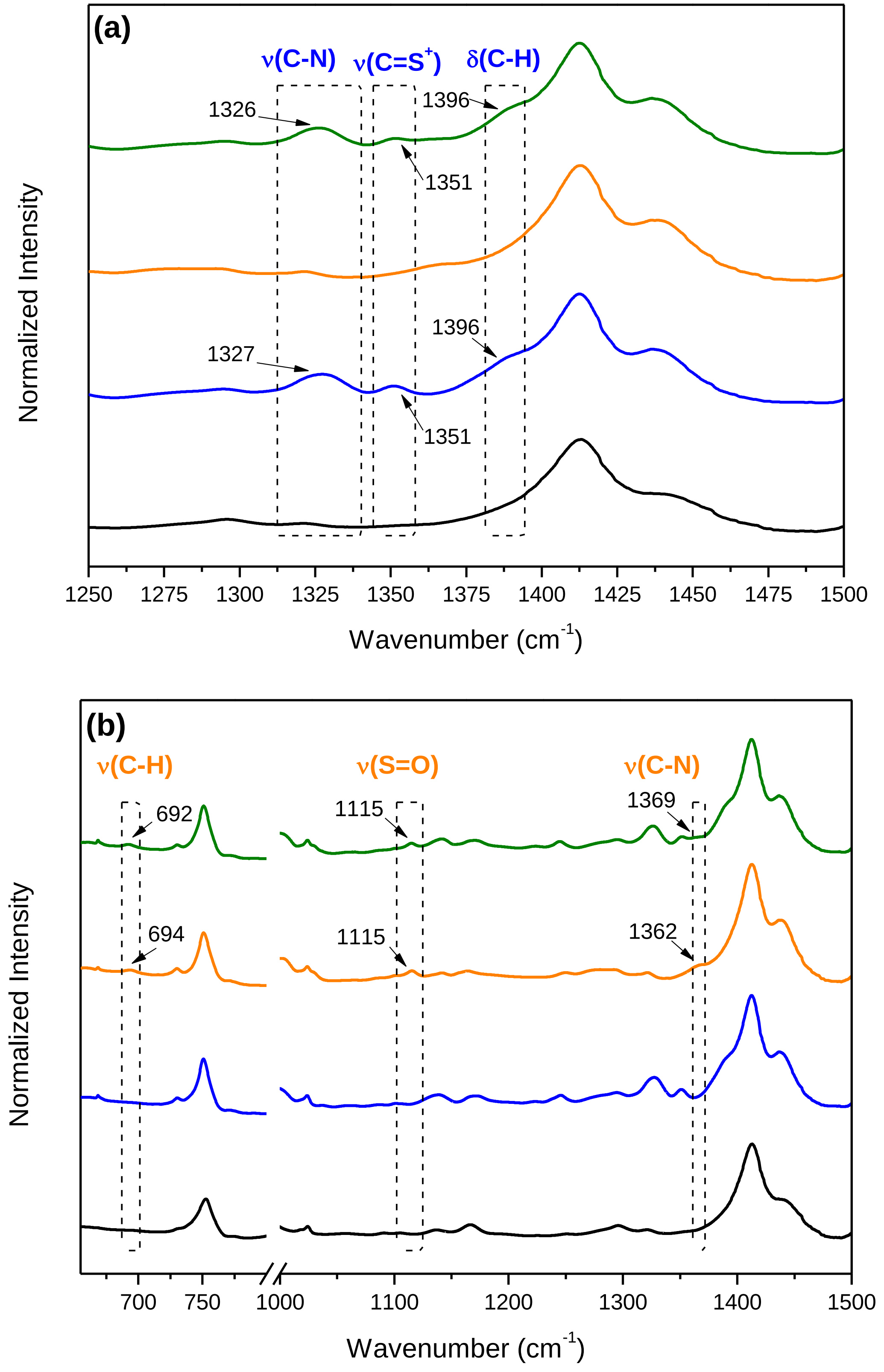

Figure S4. Characteristic FTIR peaks of **(A)** MB, and **(B)** MO dyes on MB adsorbed (blue), MO adsorbed (orange), and mixture of MB and MO adsorbed (olive) [BMIM][PF_6]/MIL-53(Al). Black line represents the FTIR spectrum of [BMIM][PF_6]/MIL-53(Al) before dye adsorption.

Table S4. Shifts in the characteristic FTIR peaks of MOF after dye adsorption (relative to the IL/MOF composite).

MOF Related Peaks (cm^{-1})	IL/MOF Composite (cm^{-1})	MB-adsorbed IL/MOF Composite (cm^{-1})	MO-adsorbed IL/MOF Composite (cm^{-1})	Mixture-adsorbed IL/MOF Composite (cm^{-1})	Assignment
658	654	**+4**	**-**	**+4**	ν_s(Al-O-Al)
687	668	**+1**	**-**	**+1**	ν_{as}(Al-O-Al)
3706	3700	**-17**	**-**	**-3**	μ_2(O−H)

Table S5. Changes in the characteristic FTIR peaks of IL after dye adsorption (relative to the IL/MOF composite).

IL Related Peaks (cm^{-1})	IL/MOF Composite (cm^{-1})	MB-adsorbed IL/MOF Composite (cm^{-1})	MO-adsorbed IL/MOF Composite (cm^{-1})	Mixture-adsorbed IL/MOF Composite (cm^{-1})	Assignment
737	731	**+4**	**-4**	**+1**	ν_{as} (PF_6) of IL's anion
837	837	**+3**	**-**	**+1**	ν_{as} (PF_6) of IL's anion
3124	3118	**-1**	**+3**	**-**	ν(C(2)−H) of IL's cation
3170	3162	**-3**	**+3**	**-**	ν_{ss}(C(4)HC(5)H) of IL's cation

Table S6. Changes in the characteristic FTIR peaks of MB after dye adsorption (relative to the IL/MOF composite).

**MB Related Peaks (cm^{-1})**	**IL/MOF Composite (cm^{-1})**	**MB-adsorbed IL/MOF Composite (cm^{-1})**	**MO-adsorbed IL/MOF Composite (cm^{-1})**	**Mixture-adsorbed IL/MOF Composite (cm^{-1})**	**Assignment**
885	-	**newly formed**	-	**newly formed**	N_{het}·······HO
1334	-	**-7**	-	**-7**	ν(C_{het}-N)
1355	-	**-4**	-	**-4**	ν($C=S^+$)
1390	-	**+2**	-	**+2**	δ(C_{het}-H)
1590	-	**+5**	-	**+5**	$ν_{ip}$(C_{het}-H)
between 1690-1710	-	**-4**	-	**-2**	ν($C_{het}=N^+(CH_3)_2$)

Table S7. Changes in the characteristic FTIR peaks of MO after dye adsorption (relative to the IL/MOF composite).

**MO Related Peaks (cm^{-1})**	**IL/MOF Composite (cm^{-1})**	**MB-adsorbed IL/MOF Composite (cm^{-1})**	**MO-adsorbed IL/MOF Composite (cm^{-1})**	**Mixture-adsorbed IL/MOF Composite (cm^{-1})**	**Assignment**
1004	-	-	**-2**	**-2**	ν($N-(CH_3)_2$)
1036	-	-	**-11**	**-10**	ν(SO_3)
1113	-	-	**+2**	**+2**	ν($C-SO_3$)
1195	-	-	**-22**	**-23**	ν(S=O)
1420	-	-	**-5**	**-6**	ν(N=N)
1444	-	-	**-5**	**-6**	ν(N=N)
1518	-	-	**-6**	**-3**	ν($C-N(CH_3)_2$)
